# X,Y, and Z: A bird's eye view on light pollution

**DOI:** 10.1002/ece3.9608

**Published:** 2022-12-15

**Authors:** Tobias Degen, Zoltán Kolláth, Jacqueline Degen

**Affiliations:** ^1^ Department of Zoology III University of Würzburg Würzburg Germany; ^2^ Department of Zoology II University of Würzburg Würzburg Germany; ^3^ Department of Physics Eszterházy Károly Catholic University Eger Hungary

**Keywords:** aeroecology, artificial light at night, ecological light pollution, evolutionary trap, nighttime migration, vertical motion

## Abstract

1. The global increase in light pollution is being viewed with growing concern, as it has been reported to have negative effects ranging from the individual to the ecosystem level.
2. Unlike movement on the ground, flying and swimming allows vertical motion. Here, we demonstrate that flight altitude change is crucial to the perception and susceptibility of artificial light at night of air‐borne organisms. Because air‐borne species can propagate through the airspace and easily across ecotones, effects might not be small‐scale. Therefore, we propose including airspace as a vital habitat in the concept of ecological light pollution.
3. The interplay between flight altitude and the effects of light pollution may not only be crucial for understanding flying species but may also provide valuable insights into the mechanisms of responses to artificial light at night in general.

## Graphical abstract

The global increase in light pollution is being viewed with growing concern, as it has been reported to have negative effects ranging from the individual to the ecosystem level. Unlike movement on the ground, flying and swimming allows vertical motion. Therefore, we propose including airspace as a vital habitat in the concept of ecological light pollution.

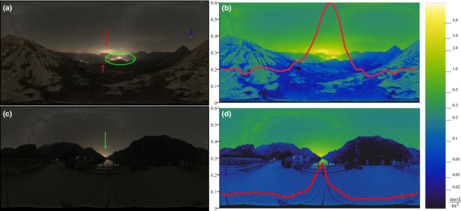

## 1 INTRODUCTION

Artificial light at night (ALAN) is so prevalent that many people from urban areas view a night sky that reveals the Milky Way with fascination. The amount of artificial light has inevitably been increasing with the continuous growth of the human population and prosperity (Kyba et al., 2017). This is a concerning trend that has generated a broad field of research and thereby a large body of scientific work demonstrating that light pollution affects humans, plants, and animals, with unforeseeable consequences for terrestrial and aquatic ecosystems (Davies & Smyth, 2018; Hölker et al., 2021). Light pollution has also been identified as a driver of broad‐scale insect decline (Owens et al., 2020) that threatens plant–pollinator communities (Knop et al., 2017).

It is therefore not surprising that the night sky is recognized as a resource worth protecting, as reflected by Darks Sky Parks' worldwide existence (Kolláth & Dömény, 2017). The term “ecological light pollution” has been coined to account for changes in natural light regimes in terrestrial and aquatic ecosystems (Longcore & Rich, 2004). However, the concept has yet to identify the airspace as a vital habitat (Chilson et al., 2017; Hölker et al., 2021; Vega et al., 2022), which is as affected by global changes in light pollution as land and water habitats (Lambertucci & Speziale, 2021) and is critically important to many living organisms (Diehl, 2013).

Artificial light at night forces nocturnal species to cope with light pollution on a large scale (Cabrera‐Cruz et al., 2018; Owens et al., 2020; Voigt, Rehnig, et al., 2018) as electromagnetic radiation, visible to humans (i.e., visible light) and insects, propagates with little distortion through the airspace (Walter et al., 2021). Therefore, the spectral distribution of visible light does not vary significantly even at moderate altitudes. However, the natural component increases and the ALAN part decreases over thousands of meters. Research has often focused on the most prominent and direct effect of ALAN: the attraction of animals to light sources (positive phototaxis) (Degen et al., 2016; Gauthreaux & Belser, 2006; Horton et al., 2019; Owens et al., 2020; Szaz et al., 2015; Voigt, Rehnig, et al., 2018). In entomology, it is often referred to as flight‐to‐light behavior (Sanders & Gaston, 2018). Potential consequences are direct mortality, for example, when birds collide with buildings, or a loss of time and energy, for example, if insects circle around a street light (Degen et al., 2016; Gauthreaux & Belser, 2006). Moreover, heavily illuminated landscapes pollute the airspace and alter the movement ecology of bats, birds, and insects (Tielens et al., 2021; Van Doren et al., 2017; Voigt et al., 2021).

In general, effects on mortality can cause indirect effects that are not limited to conspecifics or habitat borders (Degen et al., 2017). For example, in the Gulf of Mexico, tiger shark abundance was found to correlate seasonally with the migration of nonmarine bird species they consume (Drymon et al., 2019). It is important to note that migration always represents biomass transport and often occurs in airspace across ecotones (Stepanian et al., 2020), thus indicating that ALAN can affect the biomass flux, as demonstrated, for example, by the well‐known phenomenon of upstream compensation flights of mayflies (Szaz et al., 2015).

Another important aspect of light pollution is “skyglow,” that is, the diffuse illumination of the night sky (Figure 2a, red arrow and b, green arrow). With their contribution to skyglow, cities or other artificial light aggregations can be perceived from a great distance, appearing as domes of light along the horizon. It is therefore suspected that skyglow attracts animals on a large scale, as it has been, for example, observed for nocturnal migratory birds, that their density at stop‐over sites increases with proximity to illuminated areas (Horton et al., 2019; La Sorte et al., 2017; McLaren et al., 2018). However, other studies have found opposing effects suggesting repulsion by light (Cabrera‐Cruz et al., 2019, 2020; Korpach et al., 2022; Syposz et al., 2021). It is possible that seasonal, geographical, weather conditions, or taxonomic variability in bird responses to ALAN can explain these differences (La Sorte, Horton, et al., 2022; La Sorte, Johnston, et al., 2022; Weisshaupt et al., 2022). However, it is vital to note that ALAN and urbanization are strongly correlated and therefore demanding to disentangle (Korpach et al., 2022). Another instance is that ALAN, as a novel element, can create an evolutionary trap as it triggers maladaptive behavior, which reduces survival or reproduction (Schlaepfer et al., 2002; Witherington, 1997). Since ALAN triggers positive phototaxis, it has been identified as an evolutionary trap for nocturnal moths and nocturnally migrating birds (Haynes & Robertson, 2021; Robertson & Blumstein, 2019).

We aim to recognize the airspace as a distinct and vital habitat (Davy et al., 2017; Diehl, 2013; Diehl et al., 2017; Horton et al., 2016) in the concept of ecological light pollution. To this end, we discuss details on how ambient light conditions change altitudinally, and the peculiarities and limitations of light measurement along this axis. We conclude that altitude should be considered more often as a predictor than the small number of relevant publications might suggest.

## 2 FROM SURFACE TO VOLUME

### 2.1 Animals’ perspective

For various reasons, insects, birds, and bats adjust their flight altitudes by over several hundred meters to a few kilometers (Lindström et al., 2021; Norevik et al., 2021; O'Mara et al., 2021; Wainwright et al., 2020), for example, for favorable horizontal winds, feeding, protection against overheating, or orientation and navigation (Chapman et al., 2008; Dokter et al., 2013; Griffin & Thompson, 1982; Horton et al., 2016; Rydell et al., 2010).

Aside from inflight altitude adjustments and common nighttime migration, insects, birds, and bats have some other striking similarities. Across all three taxonomic groups, (a) synchronized behavior, for example, mass take‐off at dusk, has frequently been observed, presumably stimulated by changes in illumination level (Drake & Reynolds, 2012; Kunz, 1982; McLaren et al., 2018; Swift, 1980), (b) migration at high altitudes (up to a few kilometers) creates similar challenges like compensation of crosswind drift for accurate navigation (Chapman et al., 2008; Menz et al., 2022; Richardson, 1991; O'Mara et al., 2021), (c) there is evidence that they use celestial cues for navigation (Buchler & Childs, 1982; Foster et al., 2018, 2021), and (d) there is evidence that light pollution alters landscape connectivity (Degen et al., 2022, 2016; Hale et al., 2015; Korpach et al., 2022; Laforge et al., 2019).

When considering the effects of ALAN due to urbanization, positive phototaxis has been almost exclusively examined, despite the many different possible reactions to light that are even used for pest control such as repulsion via negative phototaxis (Shimoda & Honda, 2013). Especially for insects, it is presumed that the presence of light alone triggers a behavioral response. One example is the “vacuum cleaner” effect, which describes that animals are “sucked” out of the landscape by light as by a vacuum (Eisenbeis & Hänel, 2009).

However, it is well known that an organism's motivational state (e.g., foraging, roosting, migration) may matter, as indicated in the guidelines for the protection of bats (Voigt et al., 2021; Voigt, Azam, et al., 2018). Furthermore, additional predictors may play a role. For example, nocturnal migrating birds use artificial lights of urbanized areas as guiding lights only if they migrate at low altitudes with thick layers of low‐level clouds; consequently, the vertical profile of atmospheric conditions differs significantly for light scattering (Cinzano & Falchi, 2012; Weisshaupt et al., 2022). In another study, it has been shown that the flight behavior of freely moving moths gets influenced by streetlight although individuals only rarely showed flight‐to‐light behavior (Degen et al., 2022). It is noteworthy that this also applied to animals that closely passed the position of a streetlight during their flight. One conclusion of this study is that the relationship between the position of the animal and the light source, and therefore flight‐altitude, is crucial for triggering flight‐to‐light behavior. These studies demonstrate that altitude can be a predictor of the behavioral responses of animals to ALAN.

**FIGURE 1:**
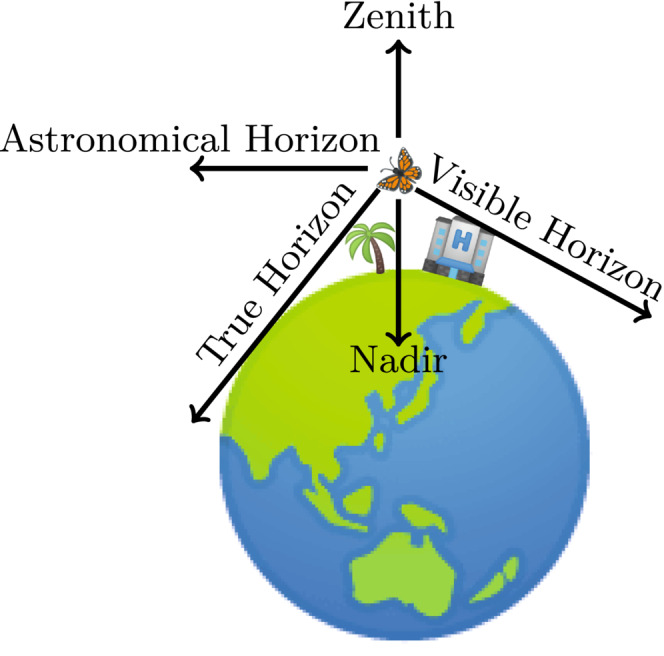
Relationship between the visible, the true, and the astronomical horizon, represented in the horizontal coordinate system with the astronomical horizon as fundamental plane and the zenith and nadir pole.

**FIGURE 2:**
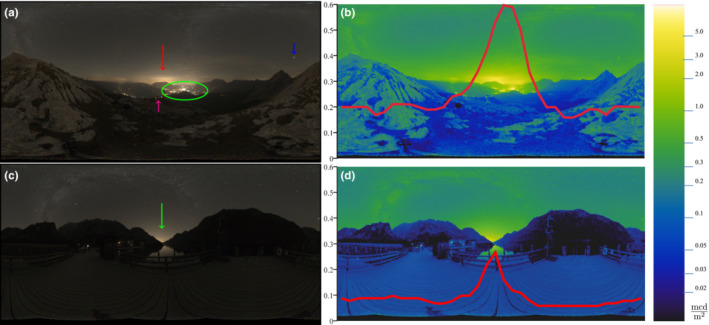
High‐resolution light pollution measurements near the Watzmann‐Hocheck (a) and (c) show the panoramic view at an elevation of 2024 m (a) and 607 m (c). Both locations have approximately the same distance to Berchtesgaden. In (a), Berchtesgaden is below the horizon (green ellipse) and in (c) beyond the horizon (green arrow) while both locations have a comparable brightness at zenith (for details see DiCaLum Report in the Appendix A). Due to the high resolution, in (a) details like, for example, the Watzmann Haus (elevation 1930 m; magenta arrow), planets like the Jupiter (blue arrow) and skyglow of Salzburg (elevation 424 m in a distance of about 30 km, red arrow) are well recognizable. Panels (b) and (d) are the associated luminance maps with the mean vertical sky brightness, excluding obscured celestial sections (like trees, hills, and buildings).

However, distance to the visible horizon and altitude are positively correlated (Gaston et al., 2021; see Figure 1). Because the visible horizon changes with altitude, ambient lighting conditions change with altitude even in landscapes without anthropogenic influence (see Figure 1). Furthermore, natural sky brightness increases from the zenith toward the horizon with an increasing gradient (Cinzano & Falchi, 2012; Duriscoe, 2013; Kwon et al., 2004). Consequently, natural sky radiation forms a symmetrical ring of light around the horizon which might be used as robust information for insects’ orientation relative to the horizon, stable enough for pitch and roll control analogous to the well‐known daytime mechanism (Srinivasan et al., 2012). However, ALAN causes a steeper gradient of sky brightness from the horizon to the zenith in the presence of moderate light pollution, especially at higher elevation sites (see Figure 2; Duriscoe, 2013). Also, it often disturbs the symmetry of the ring (see Figure 2; Duriscoe, 2013; Kolláth et al., 2021).

It is worth noting that even if the surface brightness (i.e., luminance) in clear air does not decrease significantly with altitude, the ambient light conditions change because the size of the visually emitting surface decreases with increasing distance. Finally, in the context of altitude‐dependent ambient light conditions, it should be mentioned that atmospheric conditions modulate light propagation and thus often change along the vertical axis.

### 2.2 Measurement of ecological light pollution

Measuring light pollution remains a complex challenge, which can be seen from a number of recent publications (Kolláth et al., 2021; Kyba et al., 2022; Levin et al., 2019; Masana et al., 2021). For example, investigating the impact of ecological light pollution on the behavior of living organisms is challenging since ambient lighting conditions can change rapidly and vary considerably between places (Cinzano & Falchi, 2012; Jechow et al., 2017; Vandersteen et al., 2020). Therefore, it is extremely challenging that the resolution in time and place is not a strength of today's satellite‐based remote sensing systems (Kolláth et al., 2021; Kyba et al., 2022; Levin et al., 2019; Vandersteen et al., 2020). Additionally, it is problematic that today's systems cannot detect light visible to birds, insects, and bats (Briscoe & Chittka, 2001; Cuthill et al., 2000; de Miguel et al., 2021; Winter et al., 2003).

Likewise, tricky but not systematically rooted in today's systems is that the most commonly used space‐based datasets exclude observations with moonlight or clouds (Elvidge et al., 2017; Li et al., 2020). Such exclusion of clouds could be problematic as the behavior of birds, bats, and insects can be driven by cloud cover (Cryan & Brown, 2007; Weisshaupt et al., 2022; Yela & Holyoak, 1997), and due to the exclusion of moonlight, new Moon phases are likely to be overrepresented in these datasets. This overrepresentation could be problematic because the behavior of insects, birds, and bats can also be driven by the Moon's phase or its mere presence or absence (El Jundi et al., 2015; Foster et al., 2021; Hedenström et al., 2022; Lang et al., 2006; Norevik et al., 2019; Storms et al., 2022; Warrant & Dacke, 2011). In particular, there is evidence that the lunar cycle forces the migratory activity of nocturnal birds (Norevik et al., 2019). Finally, when the Moon is 18^∘^ below the horizon, its pattern of polarized light that is used for orientation is no longer visible (Dacke et al., 2011).

Ground‐based measurements overcome two problems: nonexistent sensitivity and poor temporal resolution (Jechow et al., 2017; Kolláth et al., 2021). Unfortunately, unlike measurements taken from space, these measurements are highly laborious, especially when taken for large geographical areas (Vandersteen et al., 2020). However, an advantage of ground‐based remote sensing over space‐based remote sensing is that it can also measure light emitted from the horizon, whereas the nadir view (see Figure 1) from space measures only light emitted upwards. Vandersteen et al. stated that representative light measurements should include the horizon because it contains light stimuli in the direction of movement (Vandersteen et al., 2020). Given that air‐borne animals are not restricted to the nadir or horizontal view for orientation (El Jundi et al., 2015; Foster et al., 2018; Pritchard & Healy, 2018), high‐resolution full‐sphere photometric measurements are desirable (see Figure 2; Jechow et al., 2019; Vandersteen et al., 2020).

Here, we present two full‐sphere measurements to demonstrate the influence of altitude on the perception of ambient light conditions, as shown in Figure 2 (see Appendix A for technical details). The sites for the measurements were geographically close to each other, with an altitude range of approximately 1600 m. Thus, although they were ground‐based, they featured the main characteristics of air‐borne measurements due to the special properties of the location at the Watzmann. Moreover, we ensured that the natural components of the overall night sky brightness were comparable (see Appendix A). While ground‐based measurements in the vertical direction can only be performed at a few sites, this does not diminish their importance. The multi‐angle measurements of ALAN are essential to improve existing remote sensing retrievals and may even allow for entirely new remote sensing analyses. However, due to the laboriousness of today's multi‐angle measurement methods, they are still rare (Kyba et al., 2022; Walczak et al., 2021), and new methods to reduce the effort of acquiring the data are desirable.

## 3 RELEVANCE AND APPLICABILITY

For all flying animals, three‐dimensional movement is part of everyday life. Nocturnal animals adapted to natural light conditions where the Moon and the stars follow a predictable pattern. ALAN interferes with these natural cues, confronting the animals with modified conditions. Since ALAN nowadays affects almost every part of the world, there are no spatial limits to be defined. Thus, flying nocturnal animals must deal with non‐natural light conditions. Because organisms change their flight altitude and light changes with altitude, we believe that altitude should be considered when studying the effects of ALAN on animals. We are convinced that it can explain many previously inconclusive or contradictory results. For example, in the case of nocturnal migration, there is scientific evidence for the whole spectrum reaching from positive consequences to negative ones. For example, ALAN is a guiding light for migratory animals only at certain flight altitudes, and the maladaptive flight‐to‐light behavior (i.e., an evolutionary trap created by ALAN) of some nocturnal insects also seems to depend on the flight altitude.

Despite its influences, altitude is rarely reported as a predictor of behavioral response to light, and it is unclear whether its disregard in research is reasonable. Therefore, we encourage everyone to investigate altitude as a predictor of the impact of ALAN on air‐borne organisms, and to publish these results.

## AUTHOR CONTRIBUTIONS

**Jacqueline Degen:** Conceptualization (equal); formal analysis (supporting); funding acquisition (lead); investigation (supporting); project administration (lead); resources (supporting); writing – original draft (supporting); writing – review and editing (equal). **Tobias Degen:** Conceptualization (equal); formal analysis (supporting); investigation (lead); resources (equal); validation (lead); visualization (equal); writing – original draft (lead); writing – review and editing (equal). **Zoltán Kolláth:** Conceptualization (equal); formal analysis (lead); funding acquisition (supporting); investigation (supporting); resources (equal); software (lead); visualization (equal); writing – original draft (supporting); writing – review and editing (supporting).

## CONFLICT OF INTEREST

The authors declare no competing interests.

## Figures and Tables

**FIGURE A1:**
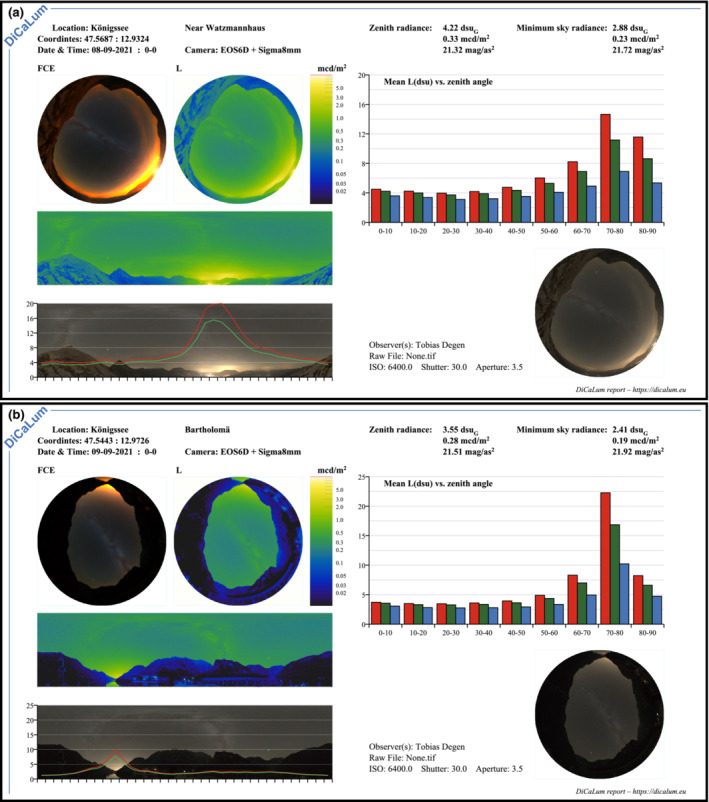
Representations of digital camera measurements after DiCaLum processing. The images at the bottom left and right show the stitched all‐sky images. FCE (top left) displays the same image with false color enhancement, which amplifies the differences compared to a normal, natural sky. The luminance (L) is presented in the standard fish‐eye projection and spherical projection. The bottom left subfigure shows the mean vertical sky radiance for green and red channel radiance in the dsu unit, excluding obscured celestial sections (like trees, hills, and buildings). The top right subfigure shows the mean horizontal sky radiance for the red, green, and blue channel in the dsu unit, excluding obscured celestial sections (like trees, hills, and buildings)

**FIGURE A2:**
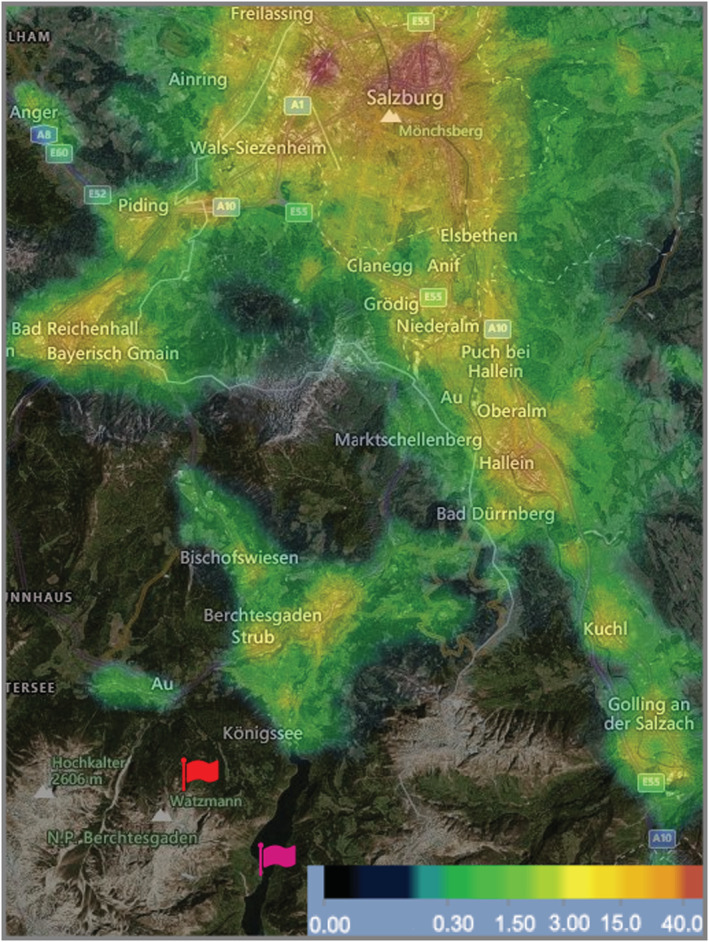
Berchtesgadener Land, light pollution map with visualized space borne Visible Infrared Imaging Radiometer Suite (VIIRS) Day/Night Band (DNB) low light imaging data (radiance 1 × 10^−9^ W cm^−1^ sr^2^). The red flag gives the measurement location close to the Watzmann Haus at an elevation above sea level of 2024 m. The magenta flag gives the measurement location at the pier St. Bartholomä at an elevation above sea level of 607 m.

**FIGURE A3:**
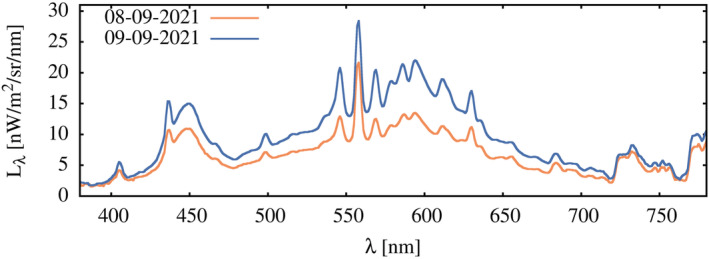
Spectra of the night sky The spectrum of the sky provides the fingerprint of the different component of sky radiance. We measured the spectra at the same location both nights (coordinates: N47.5983, E12.9850) with a Konica‐Minolta CS‐2000A spectroradiometer. At the second night, the artificial components were increased due to the increased level of humidity at the sources.

## Data Availability

The RAW digital camera measurements are published at the open‐access repository Dryad (https://doi.org/10.5061/dryad.v6wwpzh0m).

## APPENDIX A

### A.1 Full sphere heat map visualization of radiometry measures

All presented full sphere measurements consist of five images obtained with a Canon (6D) camera equipped with a Sigma lens (8 mm F3.5 EX DG CIRCULAR FISHEYE). The camera was attached to a standard three‐axis tripod. All five images were arranged approximately orthogonal to each other, with four of them facing the astronomic horizon, and one the zenith. All images were taken with the same aperture (3.5), shutter (30), and ISO number (6400).

All presented full sphere heat maps consist of five stitched heat maps. Heat maps are visualizing radiometry measures in dsu (dark sky units) or candela per square meter (luminance) units. As DiCaLum uses tiff as a container format to store heat maps, photo stitching software (e.g., Autopano Giga 4.4.2.) can be used to generate a full sphere heat map by stitching the individual heat maps to a full sphere heat map. With this full sphere heat maps (in dsu units), the presented DiCaLum reports (see Figure A1) are generated.

### A.2 Mimicked air‐borne measurements

For desired features, namely, mimicked air‐borne measurements, the crucial element for ground‐based measurements is that they should deviate as little as possible on the *x*‐ and *y*‐axis but, as in the presented case, as much as possible on the *z*‐axis between the measurement locations. This crucial element is given at the Watzmann East Face with its vertical ascent of around 1800 m. It is the longest wall in the Eastern Alps and allows perfectly to take mimicked airborne measurements. The sites for the measurements were geographically close to each other, with an altitude difference of about 1600 m; see Figure A2 for details.

To achieve sound measurements, we measured at equal weather conditions on two consecutive days. Furthermore, we took spectroradiometer measurements to ensure that natural light conditions, for example, the level of airglow, were comparable between the two nights (see Figure A3). We used a Konica‐Minolta CS–2000A spectroradiometer, as this device allows us to obtain sky spectra, even under natural sky conditions. The measurements were done with the maximum possible aperture. The exposition was set to automatic, resulting in the maximum exposure time (2 min). To improve the signal‐to‐noise ratio of the measurements, we took 5–10 spectra at the same location and direction.
